# Copy Number Variation in Chickens: A Review and Future Prospects

**DOI:** 10.3390/microarrays3010024

**Published:** 2014-02-05

**Authors:** Xiaofei Wang, Shannon Byers

**Affiliations:** Department of Biological Sciences, Tennessee State University, 3500 John A. Merritt Blvd., Nashville, TN 37209, USA; E-Mail: shannon.byers@rocketmail.com

**Keywords:** copy number variation, phenotypic variability, chicken

## Abstract

DNA sequence variations include nucleotide substitution, deletion, insertion, translocation and inversion. Deletion or insertion of a large DNA segment in the genome, referred to as copy number variation (CNV), has caught the attention of many researchers recently. It is believed that CNVs contribute significantly to genome variability, and thus contribute to phenotypic variability. In chickens, genome-wide surveys with array comparative genome hybridization (aCGH), SNP chip detection or whole genome sequencing have revealed a large number of CNVs. A large portion of chicken CNVs involves protein coding or regulatory sequences. A few CNVs have been demonstrated to be the determinant factors for single gene traits, such as late-feathering, pea-comb and dermal hyperpigmentation. The phenotypic effects of the majority of chicken CNVs are to be delineated.

## 1. Introduction

Genomes vary among different individuals of the same species, even among different cells within the same individual of multicellular organisms. Variations include differences at a single nucleotide position up to entire sets of chromosomes. Small scale variations, involving a single or a short stretch of nucleotide positions, are often discovered by sequencing. Various methods are available for detection of these small scale differences and are routinely studied for a long time. Large scale variations are visible under microscope, thus are discovered and studied routinely with microscopy during karyotyping. Variations of DNA sequences of intermediate scale, from 1,000 bp (kb) to a few million base pairs (mb), now known as copy number variation (CNV) [1,2], were ignored by researchers for some time due to the unavailability of suitable research tools. New DNA technology, especially microarray and sequencing, has permitted the convenient detection of CNVs in various genomes in the last decade, leading to the booming of CNV studies on various species, especially humans. Ongoing research into CNV will likely change the landscape of SNP-centric genome-wide association studies (GWAS) since CNV regions (CNVRs) show more inclusions and complex genetic variants than SNP sites.

Comparing among genomes of different cells within the same individual, or different individuals of the same or closely related species, DNA sequence differences can be considered as the result of nucleotide substitution, insertion, deletion, inversion, translocation, or combinations of these events. CNVs basically involve the insertion or deletion of DNA sequences, or the combination of both. An event of insertion leads to the gain of DNA segment, while an event of deletion leads to the loss of a segment. Although many CNVs involve the activity of transposable elements [3], the gain or loss of transposable elements is not considered in this category of genomic variability [4]. Because there is no convenient method to detect inversion and translocation of intermediate sizes, the scale of such structural changes is currently unknown.

In the last several years, there has been an increasing interest in the study of CNVs in the chicken. The chicken is an important farm animal species. It has served as an important protein source by providing table eggs and meat for human nutrition since its domestication in Southeast Asia over 8,000 years ago [5]. In some cultures, the chicken has also been used for ritualistic activities. Due to readily available and easy handling, the chicken has been used in fundamental biological studies, including embryonic development, genetics, immunology, oncology and virology studies. The chicken is an excellent example of the unique genome arrangement in avian species, where there are a few pairs of large chromosomes called macrochromosomes and a large number of tiny chromosomes called microchromosomes [6].

Studies of chicken CNV are driven by interests from not only the poultry science point of view, but also the basic biological perspective. Since the time when genomic tiling arrays became available for chickens [7] several years ago, a good number of studies on chicken CNV have been published. Here, we review various aspects of chicken CNVs, especially the genome-wide distribution, known phenotypic effects and areas for future research.

## 2. CNV Distribution in the Chicken Genome

The majority of chicken CNVs were detected with array comparative genome hybridization (aCGH), with which two DNA samples were labeled respectively with different fluorochromes, often cy3 and cy5, then equal amounts of labeled DNA were co-hybridized to a whole genome tiling array [8,9,10]. The hybridization signals were recorded from the tiling array using laser scanners, and the signal intensities for each probe from each DNA sample were compared [11]. The hybridization signals are supposed to have equal intensity from each of the two respectively labeled DNA samples. Sophisticated computational algorithms were then used to determine whether a region of the genome has different signal intensities. Unequal signal intensities suggest gain or loss of DNA copies in the region of genome in question. The aCGH method has been applied in studies of human and many other species [12,13,14,15].

SNP arrays have also been used to detect CNVs. Although SNP arrays are primarily designed for SNP genotyping, detection of CNV with SNP arrays is possible because abnormal hybridization occurs when a SNP probe is located in a CNV locus. Various computer programs were developed to detect CNVs from SNP genotyping data [16,17]; thus, no additional wet laboratory work is needed. As such, the detection of CNVs from SNP data added value to genome-wide association study (GWAS) with SNP arrays. This approach has been especially widely used in human population studies [18,19,20]. A few studies using SNP arrays have also been analyzed for CNV in farm animals [21,22], including chickens [23].

Reports for genome–wide detection of CNVs in chickens are accumulating (Table 1). The first study of chicken CNV with aCGH was reported by Griffin and coauthors [7] in an attempt to establish inter-species genomic rearrangement. They identified 12 CNVs between the domesticated chicken and red jungle fowl (the wild ancestor of domesticated chickens), and several other CNVs between red jungle fowl and turkey. Since this study examined only one broiler and one Leghorn chicken, the prevalence of the CNVs could not be inferred. Comparisons between the chicken and duck, and between the chicken and zebra finch revealed more inter-species CNVs along the chicken chromosome [7,24,25]. Such regions could potentially harbor CNV within the chicken because some inter-species CNVRs were also found as intra-specific CNVRs in the chicken.

**Table 1:** Current reports on genome-wide analysis of copy number variation (CNV) in chickens.

Author [ref]	Method	No. of Birds	Breed or Line *	No. of CNVRs
Griffin *et al*. 2008 [7]	aCGH	3	Red jungle fowl (1), Leghorn (1), broiler (1)	12
Skinner *et al*. 2009 [24]	aCGH	1	Red jungle fowl	32
Volker *et al*. 2010 [25]	aCGH	1	Red jungle fowl	32
Wang *et al*. 2010 [26]	aCGH	10	Cornish rock (4), Rhode island red (4), Leghorn (2)	96
Jia *et al*. 2012 [23]	SNP chip	746	White leghorn, dwarf	315
Wang *et al*. 2012 [27]	aCGH	18	Broiler (6), Leghorn (6), Chinese local breed (6)	327
Luo *et al*. 2013 [28]	aCGH	6	6_2_ (2), 7_3_ (2) and cross hybrid (2)	32
Fan *et al*. 2013 [29]	Sequencing	2	Silkie (1), local breed (1)	8,839
Crooijmans *et al*. 2013 [30]	aCGH	64	Leghorn, broiler (15 lines in total)	1,556

* Values in parentheses are numbers of birds from the breed or line.

We were the first to report intra-specific CNVRs in chickens [26]. Our study examined ten individual chickens and identified 96 CNVs corresponding to approximately 1.3% of the chicken genome, 27 of which were observed in more than one individual [26]. Since there are many well distinguished varieties of chickens, it would be of great interest if the genetic architecture that defines varieties could be elucidated. Several studies reported so far have paid attention to this issue [26,27,30]. However, each of these studies examined a small number of animals from each variety. Thus, it is uncertain regarding variety-variety specificity of particular CNVs.

Until now, Crooijmans *et al*. [30] have reported the largest number of CNVs in chickens. They identified a total of 3,154 CNVs, which were grouped into 1,556 CNVRs based on overlapping. Those CNVs were identified from 64 birds of 15 commercial and experimental lines. This study could confirm 50% of high confidence CNVRs and 23.9% of single observation CNVRs reported by Wang *et al*. [26], and 21% of the 238 CNVRs reported by Wang *et al*. [27]. Reports by others [23,28] showed additional CNVs among chickens of different variety/lines. Using whole genome sequencing approach, Fan and his colleagues reported 8,839 putative CNVs with size >2 kb, but those with size >5 kb were listed with chromosomal coordinates [29]. Taken together, currently reported chicken CNVs amount to 3,961, which aggregate to 1,876 CNVRs (see Supplement file). These CNVRs were found on GGA1-28, E64, W and Z (Table 2). The CNVRs reported on random chromosomal segments [28] in the galGal3 assembly were not counted in this statistic, due to the uncertainty about the order of probes within the random segment of the assembly. The chicken genome assembly galGal3 does not contain information on GGA29-31 and 33-38. Likely, probes on these chromosomes are represented in the unknown random chromosomal segments.

The current known chicken CNVRs encompassed 8.3% of the chicken genome, or 9.6% of the ordered assembly (Table 2). There are huge differences among chromosomes in terms of the fraction of DNA sequences involved CNVRs. On GGA1-15, CNVRs involve 5–14% of all DNA sequences. On the majority of other microchromosomes, figures are similar. Exceptions are GGA16, 25, E64, and W. GGA16 harbors the complex gene family histocompatibility proteins. Despite the fact that GGA16 is physically similar to GGA15 and GGA17, its assembled sequence is less than 4%.

In contrast to aCGH studies so far, where a small number of samples from each line/breed of chickens were analyzed, CNV identification from SNP genotyping data by Jia *et al*. [23] analyzed 746 chickens, in which 417 birds were found to harbor CNVs. Some birds were found to have no CNV in the SNP data, which is in sharp contrast to aCGH analysis. The discrepancy is understandable because (1) the 60 K SNP arrays had much lower probe density [31], while aCGH arrays contained 244,000 to 385,000 probes for the chicken genome; (2) detection of CNVs from aCGH data is based on competitive hybridization of two samples on the same array, while detection from SNP data would have to compare across different arrays. Another sharp contrast between aCGH and SNP data is the frequencies of CNVRs. Among the 315 distinct CNVs detected with SNP arrays, only five CNVs (CNVRs) had a frequency greater than 5% and none greater than 10% in the chicken population. While in aCGH studies, frequencies of 130 CNVs were greater than 10% (calculated from [30]). Such difference could result from the use of references and also probe densities. In the aCGH study by Crooijmans *et al*. [30], a red jungle fowl was used as the reference to which all other chickens were compared, while SNP chip study by Jia *et al*. [23], CNV calls ought be made in reference to the two experimental chicken lines.

Regardless of analysis with aCGH or SNP genotyping arrays, boundaries of each CNV are uncertain due to the noise of DNA hybridization and the possibility of multiple breakpoints. Comparisons between different array platforms are hindered by differences in probe locations. Many CNVs within the same CNVR, detected by different array platforms, may actually be the same allele. Another issue with hybridization detection of CNVs is the uncertainty of zygosity state. Neither aCGH nor SNP genotyping may tell whether a test sample is homozygous or heterozygous. Whole genome sequencing, on the other hand, has the potential to accurately map the breakpoints [32,33], as has been shown in the study by Fan *et al*. [29]. It is also possible that the whole genome sequencing approach can distinguish between homozygote and heterozygote. Currently, this approach faces challenges of high levels of false positive and high cost.

**Table 2:** Distribution of CNV regions (CNVRs) on chicken chromosome.

Chromosome	No of CNVRs	Total CNVR size (bp)	Assembled chromosme size (bp) in galGal3	% in CNVRs
1	364	11,375,702	200,994,015	5.66
2	311	8,125,223	154,873,767	5.25
3	180	5,540,459	113,657,789	4.87
4	159	4,609,754	94,230,402	4.89
5	123	6,862,048	62,238,931	11.03
6	93	2,262,075	37,400,442	6.05
7	66	4,203,267	38,384,769	10.95
8	51	2,875,956	30,671,729	9.38
9	54	1,443,118	25,554,352	5.65
10	43	1,412,549	22,556,432	6.26
11	57	1,955,749	21,928,095	8.92
12	34	2,906,682	20,536,687	14.15
13	43	1,362,013	18,911,934	7.20
14	41	1,136,391	15,819,469	7.18
15	35	1,551,252	12,968,165	11.96
16	1	432,778	432,983	99.95
17	22	719,234	11,182,526	6.43
18	16	1,743,280	10,925,261	15.96
19	16	524,800	9,939,723	5.28
20	31	1,596,835	13,986,235	11.42
21	16	771,271	6,959,642	11.08
22	5	267,594	3,936,574	6.80
23	18	1,223,166	6,042,217	20.24
24	12	635,342	6,400,109	9.93
25	1	2,026,539	2,031,799	99.74
26	10	998,206	5,102,438	19.56
27	18	1,438,608	4,841,970	29.71
28	11	491,924	4,512,026	10.90
E64	1	44,645	49,846	89.57
W	1	257,546	259,642	99.19
Z	43	28,708,659	74,602,320	38.48
**Total**	1,876	99,502,665	1,031,932,289	9.64

## 3. CNV and Phenotypic Variation

A CNV may affect phenotypic characteristics through various mechanisms. If a CNV is involved in protein coding, it may directly alter the protein function. If a CNV involves the regulatory region of a functional protein gene, it may alter when, where and how much of the gene is transcribed. The effect of CNV can even extend to half a megabase away [34]. It is also possible that a CNV imposes very little effect on the phenotype. While genome-wide survey seeks to provide a comprehensive map of CNVs, functional analysis provides insight into the effect of various CNVs on phenotype. In humans, the phenotypic impact of many CNVs has been demonstrated (see review by Henrichsen *et al*. [35]).

Known phenotypes associated with CNV in chickens include pea-comb [36], late-feathering on chromosome Z [37], dark brown plumage color [38] and dermal hyperpigmentation [39,40]. The functional consequences of the overwhelming majority of the chicken CNVs are yet to be revealed. As have been observed in many species, individual chickens carrying most of the CNVs appear “normal.” Because CNVs often involve large genomic regions (several kb to several mb), a large proportion of reported CNVs involve protein coding or functional RNA. Inter-species aCGH studies have shown that there are more CNVs that involve coding genes than CNVs involving solely noncoding sequences [7,24,25], regardless if the species in comparison are closely related (between turkey and chicken) or more distantly related (between chicken and duck or between chicken and zebra finch). Similarly, inter-species CNVs have similar partitions: more involve coding sequences than solely noncoding sequence.

A popular method for analysis of gene content in CNVRs is to determine enrichment of specific gene ontology (GO) terms. The list of genes in CNVRs is compared against a background gene list in a database using computational tools [41,42]. In the chicken, a striking feature is the enrichment of cytoskeletal protein genes in CNVRs [27,30], especially the keratin super family. Crooijmans *et al*. [30] suggested that such enrichment may be related to the over-representation of keratin genes in aves when compared to mammals. Gene enrichment findings of avian species are in sharp contrast to those in mammals in which enriched genes include those that respond to stimulus, antigen processing and defense [12,43]. Likely, differences are due to species-specific biology. It is also necessary to improve the background dataset so that more accurate analysis can be done.

It is common to many species that the majority of CNVs have low frequencies. Chickens are no exception. Most chicken CNVs were observed only once among the birds studied. For example, among the 96 CNVRs described by Wang *et al*. [26], 70 were detected in only one bird. Similarly, among the 3,154 CNVs reported by Crooijmans *et al*. [30], 2,210 CNVs (70%) were observed in only one bird. Some CNVs found in only one chicken in one study could be corroborated by other studies. The observed low frequencies are partly attributable to uncertainty about CNV boundaries, false negative and false positive CNV calls in aCGH. On the other hand, because some CNVs may have significant disadvantages over the individual’s phenotype, they could be under selective pressure for elimination. Thus, their frequency could not reach higher level.

**Pea-Comb Phenotype:** The pea-comb phenotype exhibits reduced size of comb and wattles in the chicken [36]. It is one of the two epistatic genes interacting with each other in classic genetics textbooks. When a chicken carries the dominant pea comb (*P*) allele at one locus and dominant rose comb allele (*R*) at another locus simultaneously, it develops walnut comb. The pea-comb is advantageous in cold climate because it reduces heat loss and makes chickens less susceptible to frost lesions [36]. Through linkage analyses using dense genetic markers and segregating families, the pea comb gene has been found within the interval containing *SOX5* on GGA1 [36,44]. The pea-comb phenotype results from a CNV: a massive amplification of a duplicated segment near evolutionary conserved non-coding sequences in intron 1 of the *SOX5* transcription factor, signifying that the duplicated expansion interferes with *SOX5* expression and the regulation of gene expression during differentiation of cells crucial for the development of comb and wattles [36]. *SOX5* encodes a member of the *SRY*-related HMG family of transcription factors and known to enrich for ECNS. It plays a role in cell fate and differentiation, skeletal development, chondrocyte development and extracellular matrix production. The interval harboring the code for pea-comb trait has been defined as 67,831,796–68,456,921 bp on chromosome 1 [36]. Interestingly, the rose comb phenotype also results from a type of structural variation. It has been demonstrated to be caused by an inversion of about 7 mb on GGA7, resulting in ectopic expression of homeodomain protein MNR2, another transcription factor [45].

**Late Feathering:** The late feathering phenotype has been widely used in commercial poultry operations for sexing of chicks at hatch. This trait is determined by a partially dominant K allele of a sex-linked locus on GGAZ. Early studies showed that the phenotype may involve an endogenous viral gene ev21. However, detailed mapping studies indicate that the K allele results from a partial duplication of the prolactin receptor gene (*PRLR*) and the sperm flagellar protein 2 gene (*SPEF2*) from the k+ allele [37,46]. This mutation causes reduced fertility and retarded development of fly feathers. Late-feathering birds not only have increased PRLR mRNA expression, but also altered mRNA levels of other genes during early development; many of them are keratin-related genes [47]. Nonetheless, transcripts of the partial duplicated *dPRLR* were found in a wide array of tissues. Its encoded protein, a truncated version of PRLR lacking a 149-aa C-terminal tail, can be potently activated by prolatin [48], suggesting the duplicated *PRLR* may be involved in a wide range of physiological activities.

**Dark Brown Plumage:** A 2011 study by Gunnarsson and coauthors found that dark brown (DB) plumage color mutation in chickens reduces the expression of black eumelanin and enhances the expression of red pheomelanin in certain parts of the plumage [38]. They demonstrated that the causal mutation factor is an 8.3 kb deletion upstream of the *SOX10* transcription start site. The SOX10 transcription factor plays a role in melanocyte development essential for melanocyte migration and survival. Deletion in this locus is thought to reduce *SOX10* expression, down-regulating expression of key enzymes in pigment synthesis like tyrosinase. Lower tyrosinase activity causes a more pheomelanistic or reddish plumage color, the characteristic feature of the DB phenotype.

**Dermal Hyperpigmentation:** Silkie chickens originate in China. These birds have unique phenotypes, including elongated feathers on the head, fluffy plumage, dark blue skin, viscera, bones and ears, feathered legs and feet, and five toes on each foot. In some cultures, people believe that the dark colored bone and skin render the chicken meat to have special therapeutical capability. The dark color in all connective tissues results from unusual melanogenesis, a condition called dermal hyperpigmentation or fibromelanosis [49]. A dominant *FM* allele was shown to be responsible for extensive pigmentation of the dermal layer of skin and the internal connective tissue [39]. The causal *FM* is an inverted duplication and junction of two genomic regions separated by more than 400 kb in wild chickens. The duplicated regions contained endothelin 3 (*EDN3*) gene that promotes melanoblast proliferation [40]. *EDN3* expression is thought to be increased in the developing Silkie embryos while melanoblasts are migrating. Elevated levels of expression are found in adult skin tissue. Comparison of four chicken breeds from Asia and Europe also displaying dermal hyperpigmentation revealed that the same structural variant regulated this phenotype across all chicken breeds. This genomic rearrangement causes a specific monogenic trait in chickens, illustrating how novel mutations with phenotypic effects have been reused during breed formation in domestic animals.

## 4. CNV and Complex Trait

Most important traits are complex, often measured in quantitative terms. This is especially true for agricultural traits such as growth rate, disease resistance, feed conversion, egg production, *etc*. In humans, there are wide interests in evaluation of the relationship between copy number variants and complex traits. Studies have demonstrated that CNVs contribute to drug response [50,51]. There are also results suggesting that an association between candidate CNVs and complex traits may be disappointing [52,53].

Studies on the relationship between CNV and complex traits in farm animals are lagging behind. In cattle, studies have suggested that CNVs may be associated with parasite resistance [54,55] and residual feed intake [56]. In swine, an attempt has also been made to associate CNVs with quantitative traits [57].

In chickens, effort was made to delineate the relationship between CNVs and Marek’s disease [28]. Marek’s disease is caused by an alphaherpesvirus belonging to the *Mardivirus* genus and is a worldwide problem in the poultry industry [58], creating substantial losses in revenue each year. Luo *et al*. [28] examined CNVs in two lines of chickens and their cross progeny that have divergent Marek’s disease susceptibility. They suggested that CNVs unique in the Marek’s disease-resistant line could be candidate conferring resistance, especially those also residing in the relevant QTL interval. They claimed that a loss CNV spanning 50 kb on GGA19 is a high confidence candidate for Marek’s disease-resistance, and that another loss CNV on uncharacterized chromosome region spanning 83.5 kb involving a general transcription factor IIi (GTF_2_I) is a high confidence Marek’s disease-susceptible candidate.

## 5. Prospects and Conclusions

Studies on CNVs are most advanced in humans and rodents. Genome-wide surveys have shown that a large proportion (up to 20%) of the human genome is copy number variable [2,59]. To date, the chicken genome has been found to have 8.3% regions being copy number variable. Although there is postulation that avian genomes have fewer CNVRs, currently, it is unlikely that we have catalogued near completion chicken CNVRs.

Many human CNVs are being studied in much broader prospects, especially regarding their involvement in human diseases. For example, the relationship between CNV and autism has been studied by many groups [60,61,62]. The involvement of CNVs in cancer is also intensively studied through genome wide analysis [63,64,65], or specifically targeted candidate analysis [66,67]. There is also significant attention to the role of rare CNVs in genetic disorders. Studies of CNV are probably at similar intensity among most farm animal species, including cattle [15,68,69,70,71,72], swine [73,74,75,76], goat [77,78], and sheep [21,79], but studies in the cattle appear more intensive.

CNV studies in chicken were not too far behind compared to those in cattle. As has been discussed above, the majority of chicken CNV studies were focused on genome-wide survey of CNVs in various breeds with aCGH or SNP arrays. Several mapping studies identified CNVs as the causal mutation for several Mendelian traits. Despite these progresses, much remains to be learned regarding chicken CNVs. First, further studies are needed to construct a more comprehensive CNV map. Current reports on chicken CNVs are limited to a small number of individuals and a limited number of breeds. Many reports indicated that most CNVs have low frequencies (<1%). It is likely that the present known CNVs are only a small portion of what exist in the chicken. Second, it is highly desirable to evaluate the phenotypic effects of chicken CNVs, especially those that may have significant outcomes. The effects of the majority of chicken CNVs are currently unknown. How these CNVs affect production, immunity growth or other important traits requires the effort of academia and the poultry industry. Since each CNV involves far greater number of DNA bases than SNPs, and many CNVs involve coding sequences, it is reasonable to believe that CNVs may contribute more importantly than SNPs to phenotypic variations. Third, heritability of chicken CNVs needs be determined. To date, no study has specifically addressed this question. Although there are sufficient reasons to believe that the majority of CNVs are transmitted in Mendelian fashion, CNV generation *de novo* does play a role [80,81,82]. This point is of special interest in poultry industry, because one of the questions in poultry breeding is how much mutations contribute to the continued improvement of breeding.

The availability of new technologies, including high density tilling array and SNP chips [83], especially massive parallel sequencing, has heralded the new era of CNV detection, fine mapping of CNV breakpoints, and perhaps zygosity status. As has been clearly demonstrated, CNVs play an essential role in certain qualitative traits. It is firmly believed that CNVs also play a role in quantitative traits. Likely, some CNVs could be the main causal factor for variations in certain quantitative traits. Studies of CNVs are in its infancy for farm animals. Development of robust and convenient CNV assays for genotyping could facilitate unveiling of genetic secrets. It could also facilitate molecular guided breeding of poultry and other farm animals.

## Supplementary Materials

Supplementary File 1
